# Prospective Assessment of Alzheimer's Disease-Like Hypometabolism Pattern in the Brain of Diabetics in Contrast to Non-diabetics on Positron Emission Tomography-Computed Tomography Images Using Fluorodeoxyglucose in India

**DOI:** 10.7759/cureus.89682

**Published:** 2025-08-09

**Authors:** Pankaj Kumar, Vikram R Lele, Md Jawed Akhtar

**Affiliations:** 1 Department of Nuclear Medicine, All India Institute of Medical Sciences, Patna, IND; 2 Department of Nuclear Medicine and PET/CT, Jaslok Hospital and Research Centre, Mumbai, IND; 3 Department of Anatomy, Indira Gandhi Institute of Medical Sciences, Patna, IND

**Keywords:** alzheimer's disease-like pattern, diabetes mellitus, fdg pet-ct, fluorodeoxyglucose, positron emission tomography-computed tomography

## Abstract

Background: Diabetes mellitus (DM) is a known risk factor for cognitive decline and Alzheimer's disease (AD), possibly due to insulin resistance and impaired cerebral glucose metabolism. Fluorodeoxyglucose positron emission tomography-computed tomography (FDG PET-CT) imaging has shown AD-like hypometabolism patterns in diabetic individuals in various global studies. However, such data is lacking in the Indian population. This study investigated the presence of AD-like hypometabolism in Indian diabetic patients compared to non-diabetic controls.

Aim and objective: This study aimed to evaluate whether the Indian diabetic patients show AD-like reduction in brain glucose metabolism, as several studies reported AD-like hypometabolism in the brain at very early stages before making a clinical diagnosis of probable AD.

Materials and methods: A prospective, observational study was conducted on 78 patients (39 diabetics and 39 non-diabetics; age range 43-87 years) at a tertiary care center. After excluding patients with a recent history of stroke, transient ischemic attack, or structural brain abnormalities, all participants underwent dedicated brain FDG PET-CT imaging just after a whole-body scan. Scans were analyzed using CORTEX-ID software (GE Healthcare, Chicago, IL, USA), comparing cerebral glucose metabolism to age-matched normative data. Regional hypometabolism was normalized to thalamic activity. Appropriate tests of significance were used, and P< 0.05 was considered statistically significant.

Results: The study included 78 patients, 39 diabetics and 39 non-diabetic controls, matched for sex (19 males and 20 females in each group). Diabetic patients had a higher mean age (66.4 ± 10.6 years). The Mini-Mental State Examination (MMSE) score was lower in diabetics (25.3 ± 2.3) than in controls (26.8 ± 1.9). About 5.1% of diabetic patients showed an AD-like pattern, and the remaining 94.9% did not show an AD-like pattern on the FDG PET-CT scan. An AD-like pattern was not seen in any patient among the non-diabetic control group. No statistically significant association was found between the AD-like pattern in the brain on FDG PET-CT and diabetes (P* = *0.494).

Conclusions: No significant incidence of "AD-like pattern" in the brain on PET-CT images using FDG was seen in this research study on the Indian diabetic populations. However, abnormal brain scans with no AD-like hypometabolism patterns possibly suggested other etiologies, likely depression. More prospective multicentric research studies on a large Indian diabetic population with age-matched non-diabetic control groups need to be explored for definite conclusions.

## Introduction

Diabetes mellitus (DM) is among the major causes of mortality and the second most frequent endocrine illness in India. Its complications are the main cause of death globally. More than 360 million individuals living in urban regions and 177 million people in rural areas in India have been diagnosed with DM, making the disease a potential epidemic [1]. According to the national epidemiological study result on diabetes carried out by the Indian Council of Medical Research (ICMR) in India, the prevalence of diabetes varied from 5.8% to 15.5% in urban areas and 3.5% to 8.7% in rural regions [2].

In India, both genetic predispositions and environmental factors are common etiologies of DM, including obesity, the gradual movement towards higher living standards, and a continuous migration to urban regions with lifestyle changes. The most prevalent non-insulin-dependent DM is characterized by obesity, insulin secretory dysfunction, insulin resistance, and excessive glucose synthesis in the liver. Type 2 DM is also linked to genetic variation. Among the common risk factors for cerebrovascular disease, DM significantly increases the risk of dementia. DM and glucose intolerance are supposed to be risk factors for Alzheimer's disease (AD), yet there is conflicting data to support this claim. There exist several potential methods by which insulin resistance could affect the metabolism of glucose in the brain [3,4].

The characteristics that differentiate AD from type 2 DM are increased prevalence with aging, a genetic predisposition, and similar pathological features in the brain and islets (pancreatic islet amyloid in type 2 DM and amyloid derived from amyloid beta protein in the brain in AD). Precursors of pancreatic and brain amyloid deposition are increasingly being linked to the pathophysiology of type 2 DM and AD, respectively [5].

As AD is a progressive neurodegenerative disorder [6] with no known etiology and few available treatments, it is crucial to accurately diagnose and differentiate it from other medical disorders resembling AD by presenting with memory impairment. Accurate diagnosis of dementia and cognitive impairment is essential for tailoring therapy and developing a care plan that enhances patient safety and minimizes the risk of preventable outcomes. Diagnosing and planning treatment early is important so that the individuals can participate in legal and financial planning and care planning.

Fluorodeoxyglucose positron emission tomography-computed tomography (FDG PET-CT) imaging plays a vital role in evaluating various neurological disorders by assessing regional cerebral glucose metabolism. It is widely used in the early diagnosis and differentiation of dementias, including AD, as well as in epilepsy, movement disorders, and encephalitis. Additionally, FDG PET is increasingly utilized to detect drug-induced neurotoxicities in humans, where altered patterns of brain metabolism can reflect neurotoxic effects of chemotherapy, immunotherapy, or other neuroactive agents before structural changes become evident. At very early stages, before making a clinical diagnosis of probable AD, several studies using FDG PET-CT imaging reported a reduction in regional cerebral blood flow and hypometabolism in the posterior cingulate gyrus (PCG), precuneus, and parietal cortices of the brain [7].

AD in an early stage can be detected on PET imaging through FDG by observing reductions in the cerebral metabolic rate for glucose (CMRglc). A few PET-CT studies using FDG reported that patients with dementia of Alzheimer's type (DAT) tend to exhibit lower CMRglc levels, particularly in the parieto-temporal, PCG, medial temporal, and/or frontal cortices. The extent of these reductions in CMRglc is linked to the severity of the condition. A decrease in CMRglc can be used to forecast the onset of DAT in individuals with mild cognitive impairment (MCI) [8]. A small number of FDG PET studies have demonstrated that decreases in CMRglc in the hippocampal formation are linked to clinical changes and can predict the shift from normal cognition to MCI and dementia [9].

Several studies have reported that individuals with metabolic syndrome are at an increased risk of developing AD compared to age- and gender-matched controls. Growing evidence suggests a strong association between AD and disruptions in both insulin signaling and cerebral glucose metabolism. This has led some researchers to characterize AD as "type 3 diabetes," reflecting its proposed nature as an insulin-resistant state of the brain [10-12]. In a study by Baker et al., a small group of individuals was examined, and the study reported that insulin resistance may affect cerebrovascular function, potentially impacting the brain's ability to utilize glucose even without clear infarction. The use of FDG PET-CT scans revealed a reduction in cerebral glucose metabolism in the parieto-temporal, frontal, and cingulate cortices. That study indicated that insulin resistance could be an indicator of AD, as in the early disease stages, the reduced glucose metabolism in the brain was associated with cognitive impairments [13].

If an AD-like pattern is often encountered in a diabetic population without cognitive impairment, it would enhance the utility of a PET-CT scan utilizing FDG to predict AD. No such study was done earlier in the Indian population. Hence, we decided to do the study to see the AD-like pattern in the brain on the FDG PET-CT scans among the Indian diabetic population.

Aim and objective

This study aimed to test the hypothesis that patients with diabetes may have an AD-like reduction in brain glucose metabolism on FDG PET-CT scans, specifically in the Indian population group.

## Materials and methods

Study design

A prospective, observational study was conducted in the Department of Nuclear Medicine of Jaslok Hospital and Research Centre, Mumbai, India, in a one-year study period after getting an informed consent form from each patient, and the study design was approved by the institute's Ethics Committee (approval number: EC/775/2014).

Study population

Sample Size

We had planned a study of independent cases and controls with one control(s) per case. The probability of exposure among controls was 0.37 in the prior study data [14]. If the true probability of exposure among cases was 0.084, we needed to study 39 cases and 39 control subjects to be able to reject the null hypothesis that the exposure rate for cases and controls is equal with a probability (power) of 0.8. The type I error probability associated with this test of the null hypothesis was 0.05. We used a continuity-corrected chi-squared statistic or Fisher's exact test to evaluate this null hypothesis. A total of 78 patients (39 diabetics and 39 non-diabetics) were included among patients with various malignancies or with infective/inflammatory pathology referred to the Department of Nuclear Medicine for PET-CT scans for their diagnostic or metastatic work-up.

Patient selection

Inclusion Criteria

Patients with documented DM were included as subjects. Sex-matched non-diabetic populations were taken as controls.

Exclusion Criteria

Patients with a recent history of stroke, transient ischemic attack, or any structural abnormalities in the brain, as seen during the PET-CT interpretation, pediatric age group patients, and pregnant women were excluded from our study.

Informed consent forms for performing the brain FDG PET-CT scan were obtained from each patient included in the study, with the Mini-Mental State Examination (MMSE) done before the scan.

Imaging protocol

Patient Preparation

All patients were injected with a range of 5-10 mCi (185-370 MBq) of FDG intravenously (weight-adjusted dose of 0.1 mCi/kg or 3.7 MBq/kg body weight) after fasting for at least six hours (hydration with water was allowed). The fasting blood sugar level was checked (measured fasting blood glucose level <160 mg/dL) with the fingertip prick just before the radio-tracer injection. A tracer uptake phase of about 60 minutes was allowed, during which the patients were made to lie on comfortable chairs in a quiet injection room without talking to avoid increased uptake in the muscles. Auditory and visual stimulations were minimized. During this period, for a whole-body PET-CT scan, patients were given oral contrast (20% mannitol, 100 ml, in 1 litre of plain water) to drink. And immediately before the PET-CT imaging, patients were asked to void and empty the urinary bladder fully.

Each patient underwent two imaging procedures: a whole-body FDG PET-CT scan performed 60 minutes after the intravenous injection of FDG, covering the region from the head to the mid-thigh, as per the original referral, followed by a dedicated brain PET-CT scan. Imaging was conducted with the patient lying on the imaging table in the supine position, lightly immobilized, especially around the head and neck. They were allowed to breathe normally during the PET and CT acquisitions. No specific breathing or breath-holding instructions were given to the patients, and scanning was performed during quiet tidal breathing.

Instrumentation

Patients were imaged on a dedicated 16-slice PET-CT scanner. The first CT scout was acquired, followed by a plain CT scan. Low-osmolar iodinated intravenous contrast was administered at the rate of 2.5 ml/sec as 1 ml per kg body weight for a whole-body PET-CT scan. Contrast images were acquired for a whole-body PET-CT scan after 50-60 seconds post-contrast injection in the helical mode (speed, 13.5 mm/rotation) from the vertex of the skull to the mid-thigh using 3.75 mm slice thickness, at 120-140 kVp and 200-300 mA. A dedicated brain PET-CT scan was acquired for six minutes in a single bed position after a whole-body PET-CT scan.

Image processing and analysis

PET images were reconstructed by standard vendor-provided iterative reconstruction algorithms using ordered subset expectation maximization (OSEM) in a 128 × 128 matrix. Emission data were corrected for scatter and random events with dead time losses using a manufacturer's software, and images were reconstructed both with and without CT-based attenuation correction.

The PET and CT images were reviewed on a workstation in all standard planes along with maximum intensity-projection (MIP) images and were analyzed visually. The software allowed the review of PET, CT, and fused data using trans-axial, sagittal, coronal, and MIP displays. Both non-corrected and corrected images were interpreted by fusing those with CT images using the supplied software.

Images were processed using a CORTEX-ID application (GE Healthcare, Chicago, IL, USA) in automatic three-dimensional stereotactic surface projection (3D-SSP) comparison to a standard age-matched normal. Scans were read by an experienced nuclear medicine physician. PET to PET regional hypometabolism normalized by the thalamus using STEP 10 and GE color maps in anterior, posterior, superior, inferior, left, and right medial and lateral projections was recorded. Hypometabolism in the PCG and precuneus with sparing of the sensorimotor cortex on the FDG PET scan of the brain was considered an AD-like pattern in this study (in AD, a peculiar regional cerebral blood flow abnormality has been reported in the PCG and precuneus even at a very early stage [7]).

Data Collection

A patient information sheet was developed to collect data from all patients through clinical history, including age, sex, fasting blood sugar level, MMSE score, PET-CT examinations, and scan findings.

Statistical Analysis

After data collection, data entry was done in MS Excel (Microsoft Corp., Redmond, WA, USA). Data was analyzed using the professional statistics package EPI Info 7.0 version for Windows (Dean AG, Sullivan KM, Soe MM. OpenEpi: Open Source Epidemiologic Statistics for Public Health. www.OpenEpi.com, updated 2013/04/06). Descriptive data were represented as mean ± SD for numeric variables and percentages and proportions for categorical variables. Appropriate tests of significance were used depending on the nature and distribution of variables, like a chi-squared test and/or Fisher's exact test for categorical variables and an independent t-test for numerical variables. Values of P less than 0.05 were considered statistically significant.

## Results

**Figure 1:**
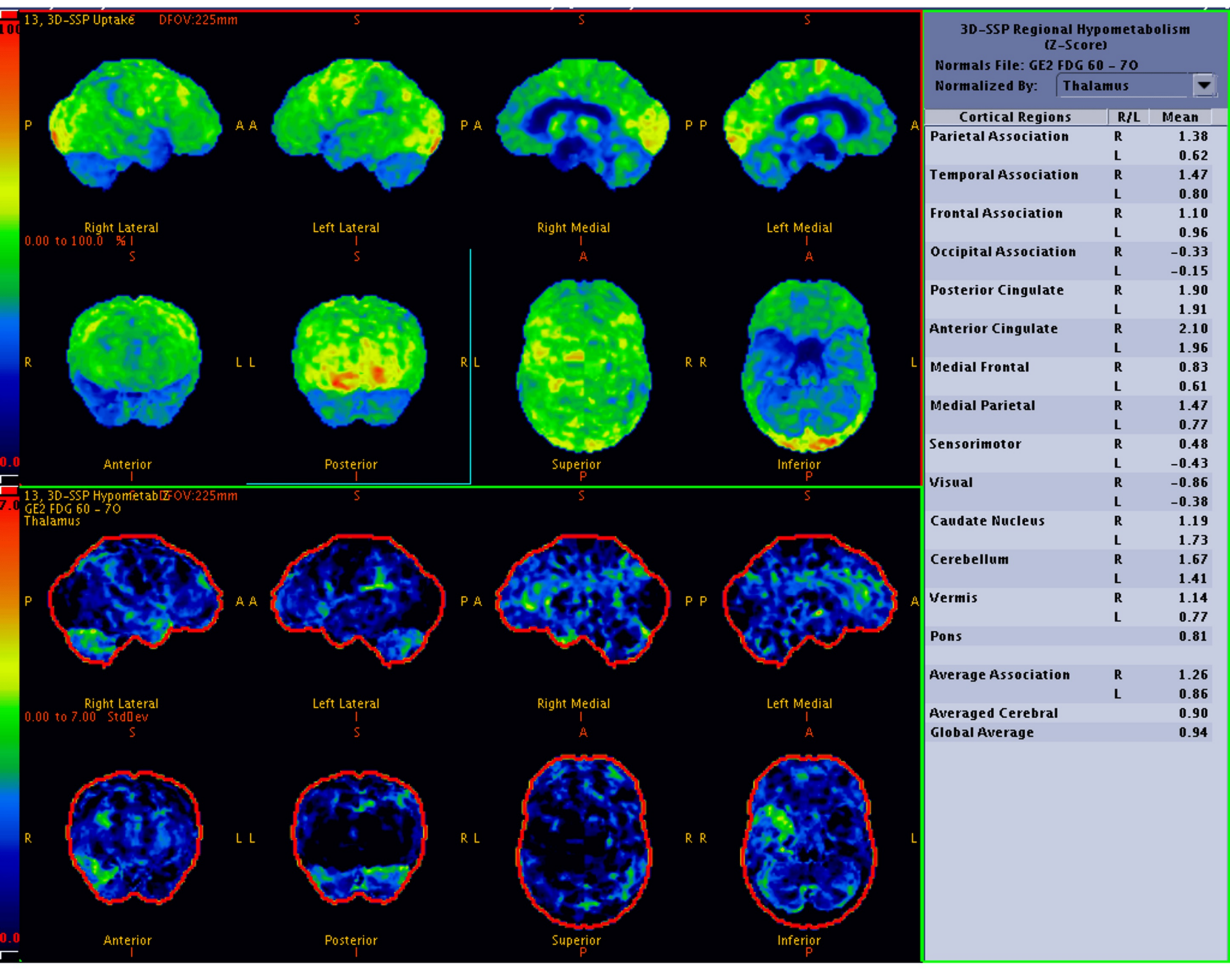
Brain FDG PET-CT scan of a 64-year-old man with carcinoma of the left lung and an eight-year history of diabetes mellitus, on oral hypoglycemic agents (FBS: 126 mg/dL; MMSE score: 21), showing global hypometabolism with sparing of the sensorimotor cortex and reduced uptake in the posterior cingulate gyrus and precuneus, features suggestive of an Alzheimer's disease-like pattern. The upper two rows show the Z-score maps of cerebral hypometabolism, while the lower two rows represent the inverse images FDG PET-CT: fluorodeoxyglucose positron emission tomography-computed tomography; FBS: fasting blood sugar; MMSE: Mini-Mental State Examination; 3D-SSP: three-dimensional stereotactic surface projection; R: right; L: left; S: superior; I: inferior; A: anterior; P: posterior

The study population consisted of 38 males and 40 females (a total of 78 patients). Thirty-nine patients (19 males and 20 females) had known DM, and 39 patients (19 males and 20 females) had no history of DM (Table 1). The mean age (in years) of patients in the diabetic and non-diabetic control groups is illustrated in Table 2. Diabetic subjects had significantly higher age in years than their non-diabetic controls (P = 0.03). The difference between their mean fasting blood sugar level was statistically significant (P = 0.0001). Diabetic subjects had significantly higher fasting blood sugar levels than non-diabetic controls (Table 3). In the present study, the MMSE score in the diabetic group was significantly lower as compared to non-diabetic controls (P = 0.002) and is represented in Table 4. Four out of 39 diabetic patients showed hypometabolism in the PCG, out of which two patients had global hypometabolism with no sparing of the sensorimotor cortex, i.e., no evidence of an AD-like pattern in those two patients; the remaining two (5.1%) diabetic patients with hypometabolism in the PCG had an AD-like pattern (Figure 1). Hypometabolism in the PCG was not statistically associated with diabetes (P = 0.115) (Table 5). Normal metabolism was noted in the PCG on FDG PET-CT scans of the non-diabetic control group.

**Table 1:** Study population: diabetics and sex-matched non-diabetic control group

Sex	Group			
	Diabetics		Non-diabetics	
	Number (n)	Percentage (%)	Number (n)	Percentage (%)
Male	19	48.7%	19	48.7%
Female	20	51.3%	20	51.3%
Total	39	100%	39	100%

**Table 2:** Mean age (in years) of the study population A P-value of <0.05 was considered statistically significant, based on an independent t-test. SD: standard deviation

Group	Number (n)	Age (in years) (mean ± SD)	P-value
Diabetics	39	66.36 ± 10.57	0.030
Non-diabetics	39	61.15 ± 10.19	

**Table 3:** Comparison of mean fasting blood sugar level in the study population A P-value of <0.05 was considered statistically significant, based on an independent t-test. SD: standard deviation

Group	Number (n)	Fasting blood sugar (in mg/dL) (mean ± SD)	P-value
Diabetics	39	130.69 ± 50.90	0.0001
Non-diabetics	39	85.00 ± 8.95	

**Table 4:** MMSE score in the study population A P-value of <0.05 was considered statistically significant, based on an independent t-test. MMSE: Mini-Mental State Examination; SD: standard deviation

Group	Number (n)	MMSE score (mean ± SD)	P-value
Diabetics	39	25.28 ± 2.34	0.002
Non-diabetics	39	26.82 ± 1.85	

**Table 5:** Comparison of metabolism in PCG in the brain on FDG PET-CT scans in the study population P-value based on Fisher's exact test. PCG: posterior cingulate gyrus; FDG PET-CT: fluorodeoxyglucose positron emission tomography-computed tomography

Metabolism in the PCG	Group				P-value
	Diabetics		Non-diabetics		
	Number (n)	Percentage (%)	Number (n)	Percentage (%)	
Hypometabolism	4	10.3%	0	0%	0.115
Normal	35	89.7%	39	100%	
Total	39	100%	39	100%	

Three out of 39 diabetic patients showed hypometabolism in the precuneus, out of which one patient had global hypometabolism with no sparing of the sensorimotor cortex, i.e., no evidence of an AD-like pattern in that patient. No hypometabolism was noted in the precuneus in the non-diabetic control group. Hypometabolism in the precuneus on the FDG PET-CT scan was not statistically associated with diabetes (P = 0.24) (Table 6).

**Table 6:** Comparison of metabolism in the precuneus in the brain on FDG PET-CT scans in the study population P-value based on Fisher's exact test. FDG PET-CT: fluorodeoxyglucose positron emission tomography-computed tomography

Metabolism in the precuneus	Group				P-value
	Diabetics		Non-diabetics		
	Number (n)	Percentage (%)	Number (n)	Percentage (%)	
Hypometabolism	3	7.7%	0	0%	0.240
Normal	36	92.3%	39	100%	
Total	39	100%	39	100%	

**Figure 2:**
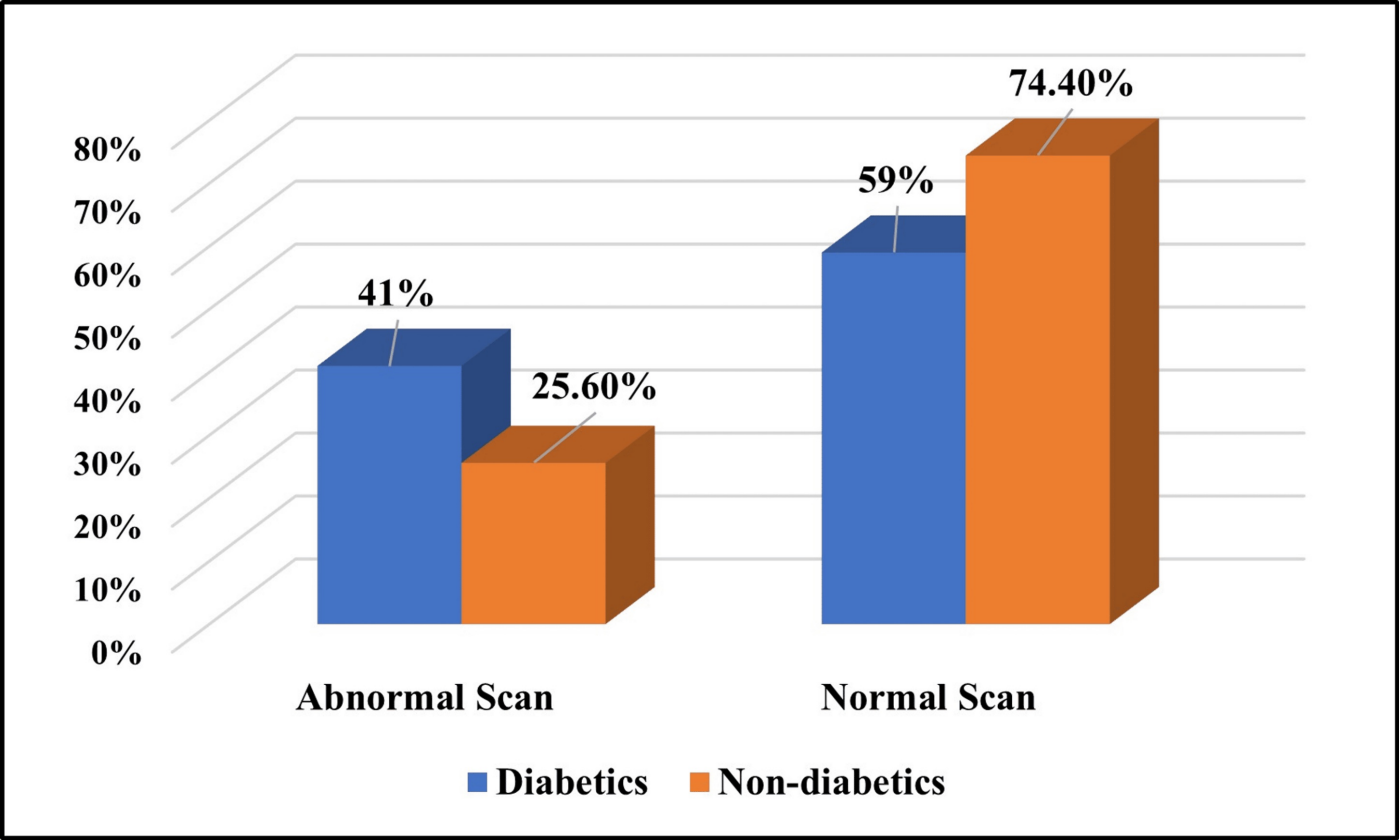
Comparison of brain FDG PET-CT scan findings in the diabetic and non-diabetic control groups showing the proportions of abnormal and normal scans FDG PET-CT: fluorodeoxyglucose positron emission tomography-computed tomography

**Figure 3:**
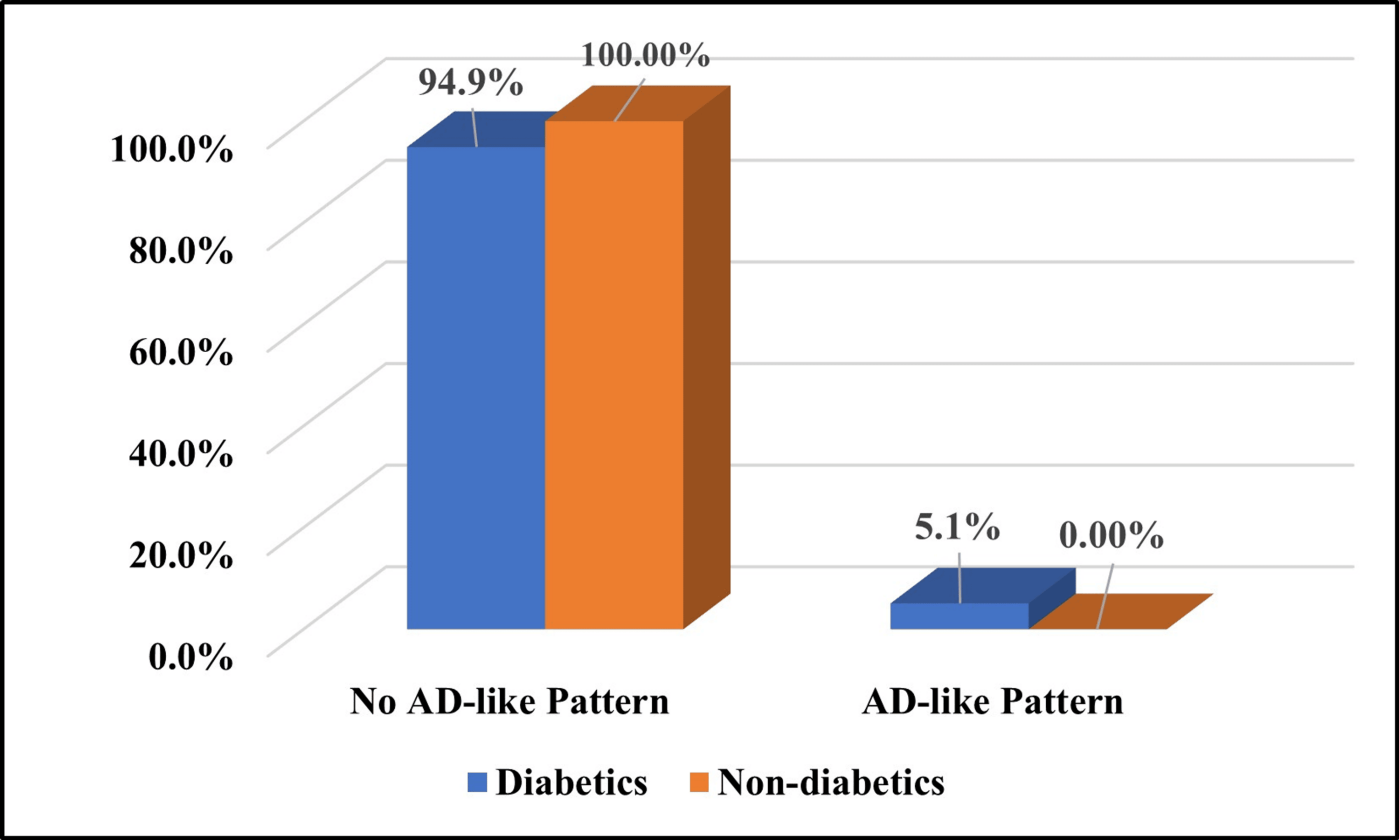
Distribution of AD-like hypometabolism patterns on FDG PET-CT brain scans in the diabetic and non-diabetic control groups FDG PET-CT: fluorodeoxyglucose positron emission tomography-computed tomography; AD: Alzheimer's disease

Out of a total of 39 non-diabetic patients, only 10 (25.6%) patients showed abnormal brain scans, but there was no evidence of an AD-like pattern on the FDG PET-CT scan (Table 7). Around 41% (16/39) of patients showed abnormal brain PET scans in the diabetic group (Figure 2), out of which only 12.5% (2/16) had evidence of an AD-like pattern on the FDG PET-CT scan (Table 8). Overall, in the diabetic group, only 5.1% (2/39) showed an AD-like pattern in the brain on the FDG PET-CT scan (Figure 3). A statistically significant association was not observed between an AD-like hypometabolism pattern in the brain on PET-CT scan in patients with diabetes (P = 0.49) (Table 9).

**Table 7:** Comparison of brain FDG PET-CT scans in the diabetic and non-diabetic control groups P-value based on Pearson's chi-squared test. FDG PET-CT: fluorodeoxyglucose positron emission tomography-computed tomography

Scan	Group				Chi-squared value	P-value
	Diabetics		Non-diabetics			
	Number (n)	Percentage (%)	Number (n)	Percentage (%)		
Abnormal	16	41%	10	25.6%	2.077	0.150
Normal	23	59%	29	74.4%		
Total	39	100%	39	100%		

**Table 8:** Comparison of abnormal brain scans on FDG PET-CT in the diabetic and non-diabetic control groups P-value based on Fisher's exact test. FDG PET-CT: fluorodeoxyglucose positron emission tomography-computed tomography; AD: Alzheimer's disease

Abnormal FDG PET-CT scan of the brain	Group				P-value
	Diabetics		Non-diabetics		
	Number (n)	Percentage (%)	Number (n)	Percentage (%)	
Abnormal but no AD-like pattern	14	87.5%	10	100%	0.508
Abnormal and AD-like pattern	2	12.5%	0	0%	
Total	16	100%	10	100%	

**Table 9:** Comparison of AD-like hypometabolism patterns on FDG PET-CT brain scans between the diabetic and non-diabetic control groups P-value based on Fisher's exact test. FDG PET-CT: fluorodeoxyglucose positron emission tomography-computed tomography; AD: Alzheimer's disease

AD-like pattern in the brain on an FDG PET-CT scan	Group				P-value
	Diabetics		Non-diabetics		
	Number (n)	Percentage (%)	Number (n)	Percentage (%)	
No	37	94.9%	39	100%	0.494
Yes	2	5.1%	0	0%	
Total	39	100%	39	100%	

## Discussion

DM remains one of the most widespread metabolic disorders globally, significantly impacting morbidity and mortality rates. AD, the leading cause of dementia, is a progressive neurodegenerative condition. Although distinct in clinical presentation, both type 2 DM and AD share common underlying pathophysiological features, such as impaired insulin signaling, chronic inflammation, and amyloid deposition, suggesting potential biological links between the two, especially in aging populations [15]. 

DM has been associated not only with cognitive decline but also with an accelerated progression towards AD-related dementia. Emerging evidence suggests that AD may involve a brain-specific form of insulin resistance, wherein insulin signaling becomes impaired in key regions responsible for memory and cognition [16].

Insulin receptors are abundantly expressed in memory-related regions of the brain. It has been postulated that enhancing insulin-receptor signaling in these areas, using certain antidiabetic agents such as insulin, thiazolidinediones, and glucagon-like peptide-1 (GLP-1) receptor agonists, may offer therapeutic benefits to individuals with AD [17]. MCI serves as an intermediate stage between normal cognitive function and dementia; however, not all individuals with MCI progress to dementia. The trajectory of cognitive and functional decline in diabetic patients with AD remains unclear and may differ from that observed in non-diabetic AD patients.

Given the progressive and largely untreatable nature of AD, early and accurate diagnosis is essential for initiating effective therapeutic interventions. A study by Baker et al. highlighted a relationship between insulin resistance and both reduced cerebral glucose metabolism and early cognitive deficits, suggesting that such metabolic changes could serve as early markers of AD [13].

In the present prospective study, 41.02% (16/39) of diabetic patients exhibited abnormal brain PET scan findings, yet only two of these showed an AD-like pattern. The remaining 14 diabetic patients demonstrated global hypometabolism without sparing of the sensorimotor cortex, findings more suggestive of depression than AD [18]. In the non-diabetic control group, 25.6% (10/39) had abnormal brain scans; however, none demonstrated an AD-like pattern. There was no statistically significant difference in the incidence of abnormal scans between the diabetic and non-diabetic groups (P = 0.15).

In terms of regional brain metabolism, hypometabolism in the PCG was observed in four diabetic patients. Among them, two had global hypometabolism without sparing of the sensorimotor cortex, thus not fulfilling the criteria for an AD-like pattern. The remaining 35 diabetic subjects, as well as all non-diabetic controls, demonstrated normal metabolism in the PCG. No statistically significant association was observed between PCG hypometabolism and diabetes (P = 0.115).

Hypometabolism in the precuneus was observed in three out of 39 diabetic patients (7.7%), including one case with global hypometabolism. The remaining 36 diabetic patients (92.3%) demonstrated normal metabolism in the precuneus. None of the non-diabetic control subjects showed hypometabolism in this region. However, the association between hypometabolism in the precuneus and diabetes was not statistically significant (P = 0.24).

Overall, only 5.1% (2/39) of diabetic patients demonstrated an AD-like pattern on FDG PET-CT scans, while none of the non-diabetic controls did. The association between DM and AD-like hypometabolism pattern on FDG PET-CT was not statistically significant (P = 0.494). These findings suggest that an AD-like pattern in the Indian diabetic population is uncommon and not statistically correlated with DM. However, the relatively high proportion (41%) of abnormal scans in the diabetic group, though non-AD-like, likely reflects other underlying etiologies such as depression.

Although some studies have reported a heightened risk of AD in diabetic patients [19,20], the present study's findings, consistent with several contradictory reports, challenge the generalization of this association. It raises the possibility that any link between DM and AD may be confined to specific, poorly defined subgroups of dementia and diabetic populations. For instance, Hassing et al. found that while DM significantly increased the risk of vascular dementia (VaD), it was not associated with AD in a population-based study of individuals aged 80 years and older [21].

Similarly, a large prospective cohort study of 3,774 Japanese-American men by Curb et al. found no significant association between diabetes (present either 15 or 25 years prior) and AD after adjusting for age and education. However, a significant link was observed between impaired glucose tolerance at baseline and VaD (P < 0.01), reinforcing the association between glucose dysregulation and vascular cognitive decline rather than AD [22].

The Baltimore Longitudinal Study of Aging (BLSA), a well-characterized prospective cohort, also found no meaningful correlation between glucose intolerance, insulin resistance, and DM and AD pathology, including amyloid beta accumulation on PET imaging [23]. Similarly, MacKnight et al., in a five-year follow-up study involving 5,574 cognitively intact individuals, reported a connection between DM and vascular cognitive impairment but no link to incident AD [24].

Postmortem studies offer additional insight. Heitner and Dickson found no increase in AD-type pathology in diabetic individuals when compared with age-matched controls [25]. Likewise, Arvanitakis et al., in their Religious Orders Study of 233 elderly clergy members, found that while diabetes was strongly associated with cerebral infarcts, it bore no relationship to global AD pathology or specific markers such as neuritic or diffuse plaques, tangles, or amyloid burden [26].

Further supporting these findings, Roberts et al. suggested that diabetes may impact cerebral energy metabolism independently of amyloid accumulation, possibly via neuronal injury mechanisms [27]. Dos Santos Matioli et al. found no increased burden of AD neuropathology in a large autopsy series of diabetic individuals [28]. Sadrolashrafi et al. similarly reported no association between type 2 DM and increased AD pathology in clinically and pathologically confirmed AD cases [29].

Current understanding suggests that DM may not directly increase AD pathology, specifically, amyloid plaques or neurofibrillary tangles, in the brain. Instead, it likely contributes to cerebrovascular disease, which may indirectly exacerbate neurodegeneration in individuals with predisposing factors such as the ApoE4 genotype [30].

Interestingly, several imaging studies have reported an AD-like hypometabolism pattern in diabetic patients, possibly due to decreased FDG uptake in regions like the precuneus. This reduction could be related to elevated fasting plasma glucose levels, potentially leading to the misinterpretation of AD in FDG PET scans, even in cognitively normal individuals with diabetes [31].

Limitations of the study

While this study provides valuable preliminary insights into AD-like brain hypometabolism patterns in the Indian diabetic population, a few limitations are noted. As a single-center study with a modest sample size, the results may benefit from validation in larger, multicentric cohorts. Although sex-matching was ensured, future studies could improve comparability by including age-matched controls. Additionally, incorporating detailed clinical parameters such as glycated hemoglobin (HbA1c) levels, serum creatinine, and antidiabetic medication profiles would enhance the depth of analysis and interpretation.

## Conclusions

In this study done in the Indian diabetic population, there was no significant association of an "AD-like pattern" in the brain on PET-CT images using FDG. However, abnormal brain scans (global hypometabolism) with no AD-like patterns found in our study were suggestive of other etiologies, like depression. Based on the findings, it is recommended that further multicentric PET-CT studies be conducted on a larger Indian diabetic population to explore the presence of AD-like hypometabolism patterns in the brain, as observed in various previous studies using FDG, which were not evident in our study. Additionally, while this study used sex-matched controls, future research should include age-matched non-diabetic Indian control groups for more accurate comparisons.
